# Knowledge, attitudes, behaviors, and serological status related to Chagas disease among Latin American migrants in Germany: A cross-sectional study in six German cities

**DOI:** 10.3389/fcimb.2022.1047281

**Published:** 2023-01-25

**Authors:** Margit Wirth, Rosa Isela Gálvez, Johannes Jochum, Ricardo Strauss, Kaja Kristensen, August Stich, Miriam Stegemann, Philipp Stahl, Karl Philipp Puchner, Jörn Strasen, Sandra Parisi, Trixi Braasch, Marion Bender, Anna Hörning, Monika Hanke, Stefan Störk, Thomas Jacobs, Michael Pritsch, Thomas Zoller

**Affiliations:** ^1^ Department of Tropical Medicine, Missioklinik, Klinikum Würzburg Mitte, Würzburg, Germany; ^2^ Protozoa Immunology, Bernhard Nocht Institute for Tropical Medicine, Hamburg, Germany; ^3^ Department of Tropical Medicine, Bernhard Nocht Institute for Tropical Medicine University Medical Center Hamburg-Eppendorf, Hamburg, Germany; ^4^ I. Department of Medicine, University Medical Center Hamburg-Eppendorf, Hamburg, Germany; ^5^ Infectious Disease Epidemiology Department, Bernhard Nocht Institute for Tropical Medicine, Hamburg, Germany; ^6^ Faculty of Life Sciences, Hamburg University of Applied Sciences, Hamburg, Germany; ^7^ Charité – Universitätsmedizin Berlin, corporate member of Freie Universität Berlin, Humboldt-Universität zu Berlin, and Berlin Institute of Health, Berlin, Germany; ^8^ Department of Infectious Diseases and Respiratory Medicine, Charité – Universitätsmedizin, Berlin, Germany; ^9^ Department of Internal Medicine, Section of Gastroenterology and Infectious Diseases, University Hospital Gießen and Marburg, Marburg, Germany; ^10^ Laboratory of Primary Health Care, General Medicine and Health Services Research, Faculty of Health Sciences, School of Medicine, Aristotle University of Thessaloniki, Thessaloniki, Greece; ^11^ Department of Pulmonary Medicine, Missioklinik, Klinikum Würzburg Mitte, Würzburg, Germany; ^12^ Department of Internal Medicine I, University Hospital Würzburg, Würzburg, Germany; ^13^ Department of General Practice, University Hospital Würzburg, Würzburg, Germany; ^14^ Department of Internal Medicine, Bundeswehr Hospital Berlin, Berlin, Germany; ^15^ Medical Department of the worldwide air ambulance Unicair GmbH, Idstein, Germany; ^16^ Department of Occupational Medicine, B.A.D, Health Center, Koblenz, Germany; ^17^ Department of Clinical Research & Epidemiology, Comprehensive Heart Failure Center, University Hospital Würzburg, Würzburg, Germany; ^18^ Division of Infectious Diseases and Tropical Medicine, University Hospital, Ludwig-Maximilians-Universität (LMU) Munich, Munich, Germany

**Keywords:** Chagas, non-endemic country, Latino, migrant, immigration, primary prevention, health initiative, knowledge

## Abstract

**Background:**

Little is known about knowledge, attitudes and behaviors concerning Chagas disease (CD) among Latin American migrants in Germany to inform public health decision making.

**Methods:**

A cross-sectional, questionnaire-based study was conducted between March 2014 and October 2019 among Latin American migrants in six cities in Germany to obtain information on migration history, socioeconomic and insurance status, knowledge about CD, potential risk factors for *Trypanosoma cruzi* infection, and willingness to donate blood or organs.

**Results:**

168 participants completed the questionnaire. The four countries with the highest proportion of participants contributing to the study population were Colombia, Mexico, Peru and Ecuador. Before migrating to Europe, the majority of the study population resided in an urban setting in houses made of stone or concrete, had higher academic education and was integrated into the German healthcare and healthcare insurance system. The majority of all study participants were also willing to donate blood and organs and a quarter of them had donated blood previously. However, many participants lacked basic knowledge about symptoms and modes of transmission of Chagas disease. One out of 56 serologic tests (1.8%) performed was positive. The seropositive female participant born in Argentina had a negative PCR test and no signs of cardiac or other organ involvement.

**Conclusions:**

The study population does not reflect the population structure at risk for *T. cruzi* infection in endemic countries. Most participants had a low risk profile for infection with *T. cruzi.* Although the sample size was small and sampling was not representative of all persons at risk in Germany, the seroprevalence found was similar to studies previously conducted in Europe. As no systematic screening for *T. cruzi* in Latin American blood and organ donors as well as in women of child-bearing age of Latin American origin is implemented in Germany, a risk of occasional transmission of *T. cruzi* remains.

## Introduction

Infection with the parasite *Trypanosoma cruzi* (*T. cruzi*) can cause Chagas disease (CD), which constitutes a major health problem in the Latin American (LA) region. According to the World Health Organization (WHO), CD belongs to the group of neglected tropical diseases which are typically associated with poverty (WHO, 2022b). CD is a chronic disease associated with disability, stigma, and a high socioeconomic burden for patients as well as for the societies in which the disease is endemic (Conteh et al., 2010; WHO, 2022b).

*T. cruzi* persists for a lifetime within host cells and the infection can cause irreversible organ damage. Organs such as the heart and gastrointestinal tract are particularly affected (Bern, 2015). Infection with *T. cruzi* remains undiagnosed in most cases. As a consequence, only 1% of patients receive treatment in the acute phase, and even less patients receive adequate medical care in the chronic phase. It is estimated that at least 6-7 million people are infected with *T. cruzi* globally, with about 38,000 new cases every year in Latin America (PAHO, 2019; WHO, 2022a).

For many decades, CD was considered an infection transmitted by contact with the faeces of an infected triatomine bug in rural areas. Migration to larger cities in the LA region led to a change in the epidemiological profile towards an emerging infection in semi-urban and urban areas (Delgado et al., 2013; Gaspe et al., 2020; WHO, 2021)

Today, due to international migration, a significant number of people infected with *T. cruzi* live in regions where the infection is not endemic. By 2009, only 4,290 *T. cruzi* cases were diagnosed in Europe; the estimated number of cases however ranges from 70,000 to 120,000 (Basile et al., 2011; Strasen et al., 2014). For this reason, the WHO strongly recommends the implementation of preventive measures to avoid transmission of *T. cruzi* infection in the European region. In some countries, such as in Spain, seroprevalence studies were conducted successfully and measures of infection control, such as screening of blood and organ donors and women of child-bearing age, were implemented and are still ongoing in people of LA origin (Monge-Maillo and L Opez-V Elez, 2017; Iglesias-Rus et al., 2019).

Estimations based on prevalence in endemic countries combined with the corresponding migrant population in Germany suggest that approximately 2,000 (range 759-3,256) migrants infected with *T. cruzi* are currently living in Germany (Strasen et al., 2014). To date, only two studies with small sample sizes have been conducted in Germany giving information on seroprevalence; a prevalence of 2% in a Latin American and a prevalence of 9% in a Bolivian migrant population were reported (Frank et al., 1997; Navarro et al., 2017 *)*.

Similar to the low level of awareness in their countries of origin, it can be assumed that people of LA origin residing in non-endemic countries are not aware of their risk for infection and their infection status (Hurtado et al., 2014; Di Girolamo et al., 2016). Moreover, they may not be aware of the fact that they could transmit the infection to others (Romay-Barja et al., 2021). Therefore, the implementation of CD screening, counseling and testing programs targeted to the population at risk could be an important public health measure to prevent *T. cruzi* transmission in Germany. This study therefore aims to examine knowledge, attitudes and behaviors towards CD in LA migrants in Germany and to explore their attitude and potential history of blood and/or organs donation to identify potential starting points for public health interventions in Germany.

## Methods

ELCiD is a German network dedicated to the identification and management of CD patients in Germany (German: “Erkennung und Lenkung von Chagas Patienten in Deutschland”, http://chagas.info). A cross-sectional study was conducted in six cities across Germany between March 2014 and October 2019. Study centers were located at Charité University Hospital in Berlin, Bernhard Nocht Institute for Tropical Medicine (BNITM) in Hamburg, Department of Infectious Diseases and Tropical Medicine at Ludwig-Maximilians University (LMU) in Munich, Department of Tropical Medicine at Missioklinik Würzburg and University Hospital in Würzburg, University Hospital in Marburg, and Kliniken Maria Hilf in Mönchengladbach. Participants meeting at least one of the following criteria were eligible: a place of birth in a country endemic to CD, residence in an endemic country for at least 10 years while under the age of 25 or a mother born in an endemic country. Endemic countries were defined according to the WHO country list (WHO, 2022a). All participants had to have their place of residence in Germany at the time of study participation. The recruitment of participants employed convenience sampling through different strategies: Individuals with LA migration background were recruited in healthcare institutions at tropical medicine outpatient departments (predominantly when attending the hospital for other pathologies or prenatal care) or at Latin American socio-cultural events. Leaflets, informative talks, and social media were used to spread information. Participants were also encouraged to invite friends and relatives to participate in the study.

After obtaining written informed consent, each participant was given a self-administered questionnaire consisting of 52 questions in their preferred language (German, Spanish or Portuguese). The questions aimed to collect data on demographics, socioeconomic background, migration history, knowledge awareness and behavior towards CD as well as willingness to donate blood and/or organs. The paper-based questionnaires were pseudonymized and imported into an electronic database located at the Comprehensive Heart Failure Center (CHFC) Würzburg. Positive response rates were used for descriptive analysis (e.g., willingness to donate blood, knowledge of CD, willingness to donate organs). Data analysis was carried out with Stata (version 15.1), Microsoft Office Excel 2019 for Mac v.16.52, and Graphpad Prism V.9.4.1. A frequency of a positive response in the range of 10% up to 50% was anticipated (Salim et al., 2010). We calculated a minimum sample size of 139 participants to achieve a precision of ±5% at a confidence level of 95% for a positive response rate of 10%.

Participants were offered testing and counselling for CD. For participants below 18 years of age, an additional version in simpler language was handed out, and consent was also obtained by parents or the legal guardian.

Blood samples were analyzed at LMU in Munich and BNITM in Hamburg. In-house ELISA and in-house IFAT assays were used for serological diagnosis of infection with *T. cruzi* (for more information on testing for *T.cruzi* in institutions in Germany, see Guggenbühl Noller et al., 2020). A blood sample was considered positive only if both, ELISA and IFAT were positive. In case of a positive serologic test result, further medical care including screening for clinical manifestations of CD and - if indicated - treatment with Benznidazole was offered. The study received ethical approval at Charité – Universitätsmedizin Berlin (EA2/209/18) and at each of the participating study sites. The study was performed according to the Helsinki declaration.

## Results

### Survey sample description

**Figure 1:**
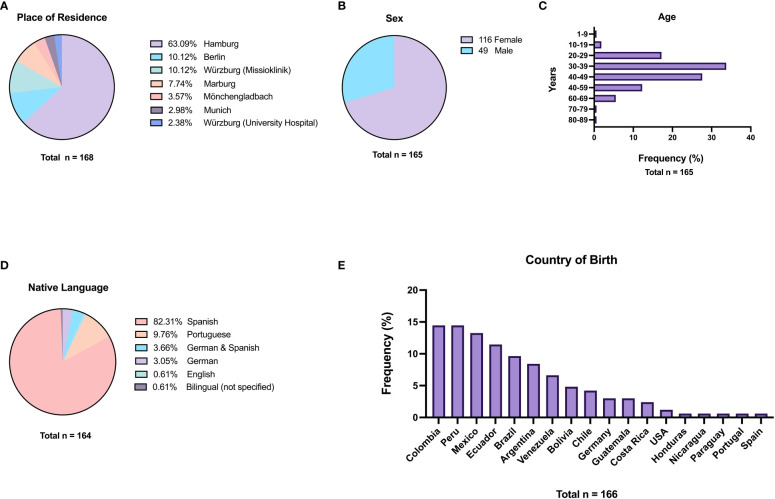
Characteristics of the study participants. **(A)** Place of residence in Germany; **(B)** Sex; **(C)** Age at time of study; **(D)** Native language; **(E)** Country of birth.

In total, 168 participants were included and answered the questionnaire. Most participants were recruited in Hamburg (63.1%), Würzburg (12.5%) Berlin (10.1%), and in Marburg (7.7%) (**Figure 1A**). Demographic characteristics of the study population are summarized in **Figures 1A–E**. Most participants were women (70.3%). The largest group of participants was aged 30 to 39 years (33.7%), followed by participants aged 40 to 49 years (27.6%). 76.7% of participating women were of reproductive age (15-49 years). Participants originated from 14 LA countries; a minimum of 10% of participants came from each of the following countries: Colombia, Peru, Mexico, and Ecuador. For most participants, the country of birth and the country where the participants were raised was identical (86.3%); this applied also to the declared country of last residence before moving to Europe (also 86.3%). 8 (4.8%) participants born in Germany, Spain, and the U.S. were included based on their mothers being born in an endemic country. Most participants (82.3%) declared to speak Spanish as their native language, and 9.7% of the participants declared to speak Portuguese as native language. Only 3.7% answered having been raised bilingually in German and Spanish. The largest group of participants migrated to Germany between 2010 and 2019 (39.1%), followed by the groups migrating between 2000 and 2009 (31.4%) and between 1990 and 1999 (19.9%) to Germany. The remaining 9.6% immigrated before 1990. Reasons for migration to Europe were marriage (32.5%) closely followed by education (30.6%), family reunification (13.8%), work (11.9%), and other reasons (11.2%).

### Socioeconomic risk factors for Chagas disease

Participants were asked for known risk factors of *T. cruzi* infection related to the setting and type of housing as well as the construction material of their last residence in LA during childhood (**Table 1**). The vast majority of participants (≥90%) lived in urban (for convenience, defined as a city of ≥10,000 inhabitants) areas in LA prior to migration to Germany as well as during childhood. Of all participants, 87.5% lived in houses constructed with stone or concrete, and only a minority lived in houses built with adobe or wood. Nevertheless, 36.9% of participants declared to have seen a kissing bug in their own house during childhood.

**Table 1:** Risk factors for Chagas disease related to location and type of housing.

Question	Choices	No. of answers	Rate of answers (%)	Total no. of answers	Total answer rate (%)
Where in Latin America did you spend most part of your childhood?	Urban	137	90.7	151	89.9
	Rural	14	9.3		
Your last residence in Latin America before coming to Europe was	Urban	141	92.2	153	91.1
	Rural	12	7.8		
What kind of material was the house made of in which you spent your childhood?	Stone/Concrete	140	87.5	160	95.2
	Adobe	9	5.6		
	Wood	8	5		
	Others	3	1.9		
Have you ever seen an insect called a kissing bug* in your own house?	Yes	58	36.9	157	93.4

*Or vinchucha, barbeiro, chirimacha, pito, chichâ.

We asked participants about their own and their parents´ educational backgrounds (**Table 2**). The participants´ highest educational degree was a university degree in 55.1% (47.5% among parents of participants) and completed professional training in 23.4% (20.6% among parents of participants). 19.6% (19.4% among parents of participants) finished secondary school and very few (1.9%, 10% among parents of participants) had finished primary school only. None of the participants answered to have no educational degree whereas 2.5% of participant´s parents had no educational degree.

**Table 2:** Socioeconomic risk factors for infection with *T. cruzi*.

Question	Choices	No. of answers	Rate of answers (%)	Total no. of answers	Total answer rate (%)
What is your highest level of education?	No degree	0	0	158	94.0
	Primary school	3	1.9		
	Secondary school	31	19.6		
	Professional Training	37	23.4		
	University degree	87	55.1		
What is the highest level of education of your parents?	No degree	4	2.5	160	95.2
	Primary school	16	10		
	Secondary school	31	19.4		
	Professional Training	33	20.6		
	University degree	76	47.5		
Have you ever heard of Chagas disease in your country of origin?	Yes	80	50	160	95.2
	No	80	50		
Does anyone in your close family suffer from/did suffer from Chagas disease?*	Yes	4	2.6	155	92.3
	No	101	65.2		
	I don´t know	50	32.2		
Do you know people in your place of residence in Latin America with Chagas disease?	Yes	21	13.1	160	95.2

*of the 4 participants, 2 indicated mother and 2 grandmothers as family members with Chagas.

### Knowledge about Chagas disease

Half of the participants had heard about CD in their country of origin before coming to Europe and 13.1% of participants knew someone with CD in their former place of residence in LA. Four participants even had close family members with CD, two of them reported having a mother suffering from CD and two reported having a grandmother with CD.

We asked participants using an open question to name the most frequent symptoms of Chagas disease (**Table 3**). Less than 5% of all study participants named at least three correct symptoms, 13.1% named two symptoms and 10.7% named only one correct symptom of CD. More than 70% gave incorrect answers or did not answer the question at all.

**Table 3:** Knowledge about Chagas disease.

Question	Choices	No. of answers	Rate of answers (%)	Total no. of answers	Total answer rate (%)
Which health problems are caused by Chagas disease? Please name the three most frequent symptoms. (Open question)	3 correct symptoms	8	4.8	n/a	n/a
	2 correct symptoms	22	13.1		
	1 correct symptom	18	10.7		
	None correct or not answered	120	71.4		
How is Chagas disease transmitted? (Multiple answers possible)	Vinchuca	92	55.8	165	98.2
	Mother/child	29	17.5		
	Sexual	2	1.2		
	Sugar cane juice	5	3.0		
	Blood transfusion	38	23.0		
	Organ transplant	19	11.5		
	Mosquito bite	15	9.1		
	Physical contact with infected person	1	0.6		
	Modes of transmission unknown	45	27.3		
Is it possible to have Chagas disease without having symptoms?	Yes	65	42.2	154	91.7
	No	8	5.2		
	I don´t know	81	52.6		

**Figure 2:**
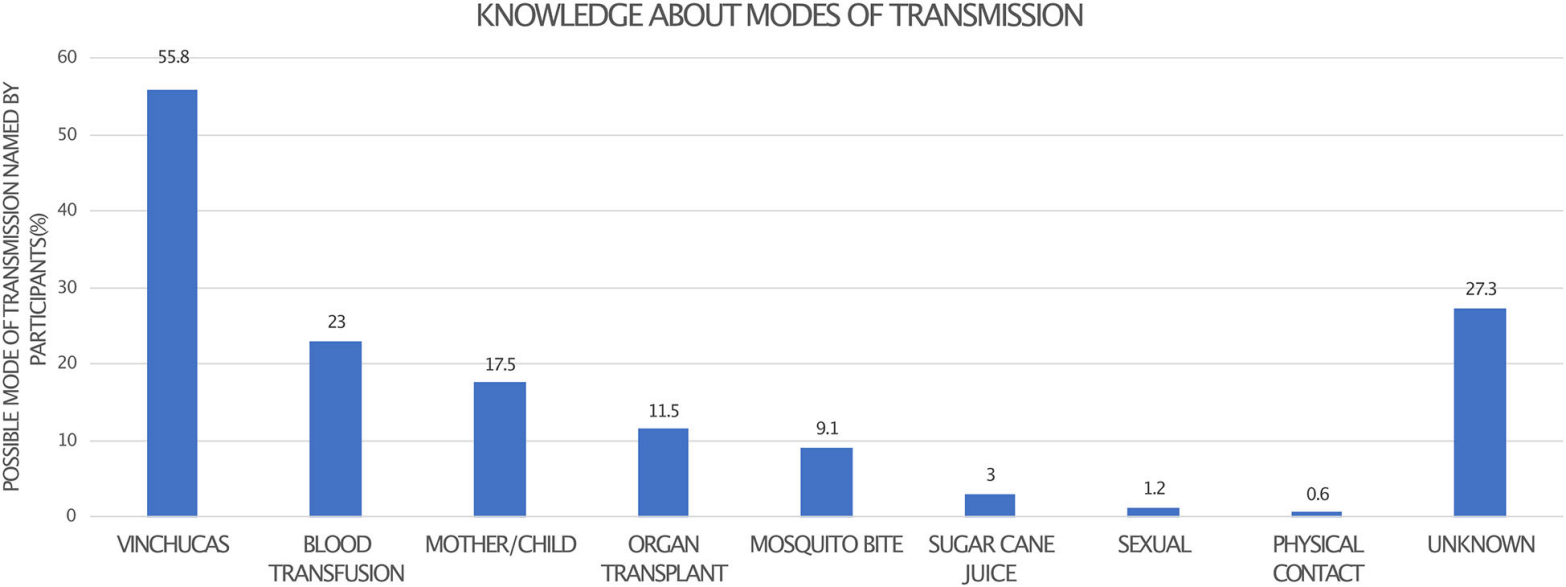
Participants were asked to name all correct modes of CD transmission.

We then offered several pre-specified correct and incorrect answers for CD transmission (see **Supplementary Material**) and participants were asked to mark the correct modes of transmission. 55.8% correctly identified transmission *via* a vector, 23% transmission *via* blood transfusion, and 17.5% congenital transmission from mother to child. Only 3% of participants considered oral infection by consuming contaminated sugar cane juice as a correct option; for details see **Figure 2**. 42.2% of respondents confirmed correctly that affected persons can be without symptoms despite being infected with *T. cruzi*.

### Attitude towards blood and organ donation

66% of participants are willing to donate blood in the future and 55.1% are willing to donate organs. 43 participants (26.9%) had donated blood previously, 14 (32.6%) of them in Germany. 11 participants (6.9%) had received a blood transfusion previously, and six of those received the transfusion in a country endemic to CD (**Table 4**).

**Table 4:** Risk of transmission in non-endemic areas.

Question	Choices	No. of answers	Rate of answers (%)	Total no. of answers	Total answer rate (%)
Have you ever received a blood transfusion?	Yes	11	6.9	160	95.2
	No	149	93.1		
Have you ever donated blood?	Yes	43	26.9	160	95.2
	No	117	73.1		
Country of previous blood donation	Argentina	4	10	40	93.0*
	Brazil	2	5		
	Chile	2	5		
	Colombia	3	7.5		
	Ecuador	1	2.5		
	Germany	11	27.5		
	Guatemala	1	2.5		
	Mexico	7	17.5		
	Peru	2	5		
	Spain	2	5		
	USA	1	2.5		
	Venezuela	1	2.5		
	Germany and LA	3	7.5		
Year of blood donation	1970-1979	1	3.2	31	72.1*
	1980-1989	0	0		
	1990-1999	6	19.4		
	2000-2009	8	25.8		
	2010-2019	16	51.6		
Are you willing to donate blood in the future?	Yes	107	66.0	162	96.4
	No	33	20.4		
	I don´t know	22	13.6		
Are you willing to donate organs after death?	Yes	93	57.1	163	97.0
	No	36	22.1		
	I don´t know	34	20.9		

*Answer rate of all participants that replied “Yes” to blood donation.

### Serological status and prior Chagas treatment

Only 13 participants (8%) stated to have been tested for CD before, with two of them reporting a positive result (details see **Table 5**). One participant indicated to have received Chagas therapy before. Of all 168 participants, 56 (33.9%) consented to serological testing for CD after completing the questionnaire. One participant was found to have a positive test result. The seroprevalence of infection with *T. cruzi* was 1.8% in our study population. The participant with a positive test was a female born in 1988 in Argentina to a mother known to be seropositive. She never had lived in a rural area and reported to have never seen a triatomine bug or having received a blood transfusion; congenital transmission is the most probable way of infection in this case. The participant who tested positive was asymptomatic, had a negative PCR result, and no evidence of CD in ECG and echocardiography. She had never donated blood before. She was aware of her serostatus before the test was done and stated that she had been looking unsuccessfully for specialized medical care as she felt that her German family practitioner did not take her condition seriously.

**Table 5:** Medical history.

Question	Choices	No. of answers	Rate of answers (%)	Total no. of answers	Total answer rate (%)
Have you ever been tested for Chagas disease?	Yes	13	8.0	163	97.0
	No	150	92.0		
Year of previous test	1990-1999	3	25	12	7.1
	2000-2009	3	25		
	2010-2019	6	50		
Result of previous test	Positive	2	18.2	11	6.5
	Negative	9	81.8		
Have you ever received treatment against Chagas disease?	Yes	1	5.6	18	10.7
	No	17	94.4		
Do you suffer from heart disease?	Yes	6	3.7	161	95.8
	No	155	96.3		
If you suffer from heart disease, what is the diagnosis?	Hypertension	1	16.7*	3	1.8
	Myocardial infarction	2	33.3*		
Do you suffer from gastrointestinal disease?	Yes	20	13.1	153	91.1
	No	133	86.9		
If yes, what are your symptoms or which diagnosis was given to you? (open question, multiple answers possible)	Chronic constipation	1	5**	20	11.9
	Diarrhea	1	5**		
	Esophageal hernia	1	5**		
	Fatty liver	1	5**		
	Flatulence	1	5**		
	Food intolerance	1	5**		
	Fructose intolerance	1	5**		
	Gall stones	1	5**		
	Gastritis	6	30**		
	Gastroenteritis	1	5**		
	Irritable bowel syndrome	3	15**		
	Lactose intolerance	2	10**		
	Pain in the mouth/stomach	1	5**		
	Reflux	5	25**		
	Stomach problems	2	10**		

* Of all participants that suffer from heart disease.

** of all participants that suffer from gastrointestinal disease.

### Access to healthcare in Germany

More than 90% of participants were members of the German health insurance system (statutory or private health insurance), and 4.2% had student insurance. Only 2.5% were insured with foreign insurance and 2.5% had no health insurance (**Table 6**).

**Table 6:** Access to medical care in Germany.

Question	Choices	No. of answers	Rate of answers (%)	Total no. of answers	Total answer rate (%)
Do you get sufficient medical care in Germany?	Yes, there are no problems to get sufficient health care	134	84.3	159	94.6
	Yes, but there are some problems to get health care	16	10.1		
	No, there are severe problems to get health care	9	5.7		
What kind of health insurance do you have in Germany?	German health insurance (statutory/private)	148	90.8	163	97.0
	Health insurance for students	7	4.3		
	Foreign insurance	4	2.5		
	None	4	2.5		

The vast majority of respondents (84.3%) did not report difficulties obtaining adequate healthcare in Germany, 10.1% reported that they encountered minor problems and 5.7% said they experienced high barriers to access medical care.

## Discussion

This cross-sectional study among people living in Germany with a history of migration from countries where CD is endemic presents information on knowledge, behavior, social determinants, and serological results relevant for understanding the epidemiology and potential risk factors for *T. cruzi* infection in Germany.

In general, the results indicate that the population in Germany at risk for infection with *T. cruzi* may differ in many aspects from the typical population at risk in endemic countries. The majority of participants come from urban areas and are either well educated or come for academic or professional purposes to Germany. Most participants grew up in houses with a low risk for vector infestation. Our results confirm earlier observations from a cohort of Bolivian migrants in Munich where a very similar population structure in terms of education and socioeconomic status was found (Navarro et al., 2017). Even though the representativeness of these two available studies in Germany is still limited, the data nevertheless indicate that assumptions made e.g., on the prevalence of *T. cruzi* infection in the home countries of migrants may not apply to the corresponding migrant population in Germany. This may have implications, particularly for the frequently used method of estimating prevalence in non-endemic countries by extrapolating prevalence in endemic countries to the migrant population in non-endemic countries (Gascon et al., 2010; Strasen et al., 2014; Requena-Méndez et al., 2015). As the risk profile for this poverty-related infection among LA migrants in Germany was lower than the average in the respective LA countries, there is a risk for over-estimating prevalence in non-endemic settings. Regarding screening activities for *T. cruzi* infection in Germany, these results show that - besides CD prevalence in the country of origin - an evaluation of individual risk factors (e.g., exposure to vectors in childhood, housing status and setting, individual socioeconomic status) might be more important than using migration history from LA alone as the dominant criterion assessing the individual risk for infection with *T. cruzi*.

Population-representative studies accurately determining seropositivity for *T. cruzi* infection in non-endemic countries do not exist, but accurate data would be of outstanding importance for making public health decisions, e.g., for ante- and perinatal medical care or screening in blood and organ donors. The best available data comes from cohorts of LA blood donors in Spain where seroprevalence rates between 0.05% and 1.38% among LA migrants were observed, which is slightly less than the average of the calculated country-specific prevalence in Spain obtained by extrapolation from endemic country prevalence (average 2.1%, range 0 - 10.9%) (Gascon et al., 2010). A recent study analyzing laboratory data from German test centers found a positivity rate of 1.4% among all tested individuals (not all persons tested were of LA origin) (Guggenbühl Noller et al., 2020). Together with the only other study in Germany (Frank et al., 1997) giving a prevalence estimate of 2%, data from this study adds further information to support the hypothesis that an overall prevalence among LA migrants in Germany between 1 – 2% can be assumed.

The majority of respondents in our study were willing to donate blood. 14 (8.3%) of them had already donated blood in Germany. Unfortunately, the German blood transfusion system is not yet prepared to prevent Chagas transmission. Currently, the following policy, which is in line with the European Commission guidelines on the quality and safety of blood and tissue banks, is in place: If a donor is aware of being infected with *T. cruzi*, he/she will be automatically excluded from donation (Bundesärztekammer et al., 2021). However, if the donor is not aware of an infection and no routine screening for CD is performed at the blood bank, a transmission is not prevented *via* blood donation in Germany. Supported by the data from this and other European studies, this practice should be reconsidered and blood donors of LA origin should actively be offered screening for infection with *T. cruzi*.

Local access to healthcare is a key determinant of health for migrants. Although access to healthcare varies among the specific countries and immigrant populations, it is a common pattern that especially undocumented migrants experience very limited access to healthcare. In our group of participants, more than 80% declare to have sufficient access to the German medical system, while only 5.7% reported having problems.

Regarding the congenital transmission of CD in non-endemic regions, the most well-documented data comes from the U.S. and Spain. It is estimated that there are approximately 40,000 women of childbearing age infected with CD in the U.S. while the risk of transmission ranges from 1 - 5%. Thus, there may be as many as 300 congenital cases in the United States per year (Bennett et al., 2018; Edwards et al., 2019). In Europe, similar transmission rates in Spain and Italy (Rodari et al., 2018; Basile et al., 2019) are reported. There are no preventive measures in place to avoid congenital transmission in Germany until now. In our study, we show that only 21.3% (17 out of 80) of women of childbearing age were aware of the possibility of vertical transmission. This finding, in combination with lacking routine screening measures in pregnancy, indicates a potential risk for Chagas vertical transmission in Germany.

Overall, our results show that most Latin Americans who participated in the study lacked knowledge about CD, particularly about how it can be transmitted despite the participants’ high socio-economic and education status. Taking further into account that information about CD could have been conveyed prior to study participation, we must assume that knowledge about CD in the general LA migrant population is even lower than described here. The lack of information in the study population can be explained by the fact that CD is a neglected and stigmatized disease in endemic countries. Most participants were curious after completing the questionnaire and wanted to learn more. They received comprehensive information about CD after study participation.

This cross-sectional study had limitations. The sample size is limited and the study team did not have resources to conduct a study representative of Germany’s entire LA migrant population. Convenient sampling was used, which likely caused a sampling bias. In particular, participants with a lower socioeconomic status could have experienced higher barriers and had less opportunity to take part in this study. Participants recruited at healthcare institutions probably had already less problems accessing the healthcare system than the average LA migrant population. Moreover, a network bias (i.e., persons of the same socioeconomic status may have promoted participation mostly to other people with similar status) can be assumed.

In conclusion, migrants from LA living in Germany who participated in this study had a high socioeconomic status, a high level of education, and were well integrated into the German society. Despite a potential bias in recruitment, the results indicate that the LA migrant population in Germany may differ from the population structure in their respective home countries. Risk factors for infection with *T. cruzi* may not be evaluated by migration status alone; an individual assessment of known risk factors of exposure in their respective home countries and the risk of vertical transmission should be equally considered. Despite a high level of education, knowledge about CD was still poor. Serological prevalence of infection with *T. cruzi* in this non-representative study was comparable to previous and other European data but highlights again the need for improved guidelines and practices particularly regarding ante- and postnatal care as well as blood transfusion and organ transplantation services.

## Data availability statement

The original contributions presented in the study are included in the article/**Supplementary Material**. Further inquiries can be directed to the corresponding author.

## Ethics statement

Written informed consent was obtained from the individual(s) for the publication of any potentially identifiable images or data included in this article.

## Author contributions

MW, AS, MP and TZ conceived the study. All authors contributed to data collection. MW, RG analyzed the data; MW, RG and wrote the manuscript and TZ guided the writing process. All authors contributed to reviewing the final manuscript.
