# Supplementary material for: Knowledge, attitudes, behaviors, and serological status related to Chagas disease among Latin American migrants in Germany: A cross-sectional study in six German cities
Source: Front Cell Infect Microbiol. 2023 Jan 25;12:1047281. doi: 10.3389/fcimb.2022.1047281 (PMC9905718; doi:10.3389/fcimb.2022.1047281)
Supplement: Supplementary file 1 [file DataSheet_1.pdf]

Número de participante (llenado por el responsable del estudio)

ELCiD (Erkennung und Lenkung von Chagas-Patienten in Deutschland (ELCiD)/ Querschnittstudie)

Proyecto alemán de Chagas
Cuestionario sobre la enfermedad de Chagas en Alemania

Estimado(a) Participante,
Uno de los objetivos del estudio de Chagas, es mejorar la atención médica en Alemania para inmigrantes o personas con riesgo para la enfermedad de Chagas. Para esto requerimos información acerca de su persona y de los conocimientos que Usted tiene sobre el Chagas. En el contexto de nuestra oferta de asesoramiento y de la prueba de Chagas, le solicitamos contestar algunas preguntas.
Su información se mantendrá estrictamente confidencial y estrictamente por separado de su información personal (por ejemplo, nombre, fecha de nacimiento). La evaluación se realiza exclusivamente de manera estadística. De esta manera ninguna conclusión sobre datos y personas individuales será posible.
¡Le agradecemos su ayuda!

1. Su sexo es ☐ **masculino** ☐ **femenino**

2. Año de nacimiento _______________

3. País de nacimiento: ________________ **4. Cuidad de nacimiento:** ____________________

5. Nacionalidad: ______________________ **6. Idioma materno:**___________________

7. ¿En qué año llegó a Europa? ___________________________

8. ¿Cual fue la razón por su traslado/ mudanza a Europa?

☐ trabajo ☐ estudios ☐ matrimonio/pareja ☐ familia vive en Europa
☐ otras razones, que son (9) ____________________________________

10. ¿En qué país pasó la mayor parte de su infancia? ________________________

11. ¿En qué ciudad pasó la mayor parte de su infancia? ____________________________

12. ¿En qué país/ ciudad fue su último domicilio antes de trasladarse a Europa?

país: _______________________________ ciudad/región (13) :_________________________

14. ¿En qué lugar de Latinoamérica pasó la mayor parte de su infancia?

☐ En la ciudad (más de 10.000 habitantes)
☐ En el campo (menos de 10.000 habitantes)

15. Su último domicilio en Latinoamérica fue....

☐ En la ciudad (más de 10.000 habitantes)
☐ En el campo (menos de 10.000 habitantes)

16. ¿De qué material fue construida la casa en que pasó su infancia?

☐ piedra/concreto/ cemento ☐ adobe/ barro ☐ madera
☐ otros materiales de construcción

17. ¿De qué material fue construida la casa de su último domicilio en Latinoamérica?

☐ piedra/concreto/ cemento ☐ adobe/ barro ☐ madera
☐ otros materiales de construcción

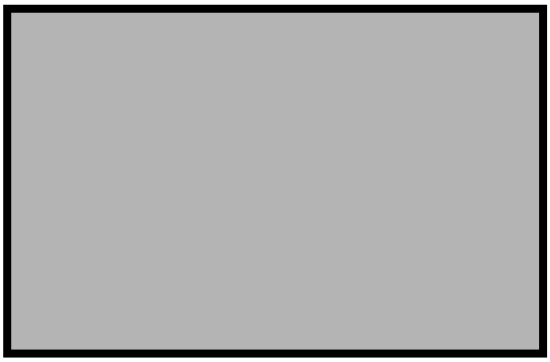

18. ¿Ha visto alguna vez en su domicilio un insecto llamado chinche (o vinchuca, barbeiro, chirimacha, pito, chichâ...)?

☐ sí ☐ no

19. ¿Cuántos hijos tiene? ___________________________________

20. ¿Cuál es su máximo nivel de educación?

☐ Ningún título escolar ☐ primaria ☐ secundaria ☐ formación técnica

☐ formación universitaria

21. ¿Cuál es el máximo nivel de educación de sus padres?

☐ Ningún título escolar ☐ primaria ☐ secundaria ☐ formación técnica

☐ formación universitaria

22. ¿Ha escuchado de la enfermedad de Chagas en su país?

☐ Sí ☐ no

23. ¿Cuáles son las molestias que la enfermedad de Chagas puede causar?
Por favor mencione las tres más frecuentes

1.____

2.____

3.____

24. ¿Cómo se transmite la enfermedad de Chagas?
(por favor marque todas las maneras de transmisión que conoce)

☐ **Por un chinche (vinchuca)**
☐ **Por relaciones sexuales**
☐ **Por una transfusión de sangre**
☐ **Por una picadura de zancudo**
☐ **Por un transplante de órganos**
☐ **Por beber jugo de caña**
☐ **Durante el nacimiento de la madre al bebé**
☐ **Por contacto físico con personas infectadas**
☐ **No conozco las maneras de transmisión**

25. ¿Hay personas que se sienten bien aunque están infectadas con la enfermedad de Chagas?

☐ sí ☐ no ☐ no sé

26. ¿Alguien de su familia cercana padece/padeció de la enfermedad de Chagas? (padres, abuelos, hermanos, niños)

☐ Sí, es mi (27) ______________________________ ☐ no ☐ no sé

28. ¿Conoce a personas de su lugar de residencia en Latinoamérica con la enfermedad de Chagas?

☐ sí ☐ no

29. ¿Podría imaginarse donar sangre en el futuro?

☐ sí ☐ no ☐ no sé

30. ¿Ha donado sangre alguna vez?

☐ sí, en el siguiente país (31) ______________________________ en el año (32) ___________

☐ no

33. ¿Ha recibido una transfusión de sangre alguna vez?

☐ sí, en el siguiente país (34) ______________________________ en el año (35) ___________

☐ no

36. ¿Podría imaginarse donar sus órganos después de su muerte?

☐ sí ☐ no ☐ no sé

37. ¿Le han hecho una prueba de Chagas alguna vez?

☐ Sí, en el año (38) _________________________

(39) el resultado dio ☐ positivo ☐ negativo

☐ no

40. Si el resultado fue positivo, ¿recibió algún tratamiento para la enfermedad de Chagas?

☐ sí, en el siguiente país (41): ____________________en el año (42) ______________________

con el siguiente medicamento (43) ________________________________

☐ no

44. Si ha padecido de la enfermedad de Chagas alguna vez, ¿cuáles molestias ha tenido/tiene?

1. ____

2. ____

3. ____

45. ¿Padece de una enfermedad al corazón?

☐ sí ☐ no

46. Si padece de una enfermedad al corazón, ¿cuáles molestias tiene?

1.____

2.____

3.____

47. Si padece de una enfermedad al corazón, ¿cuál es el nombre de su enfermedad?

1.____

2.____

3.____

48. ¿Padece de una enfermedad al tracto digestivo (estómago, esófago, intestino)**?**

☐ sí ☐ no

49. Si padece de una enfermedad al tracto digestivo, ¿cuáles molestias tiene?

1.____

2.____

3.____

50. Si padece de una enfermedad al tracto digestivo, ¿cuál es el nombre de su enfermedad?

1.____

2.____

3.____

51. ¿Usted personalmente siente que su atención médica en Alemania es suficiente?

☐ sí, ya que no tengo problemas con el acceso a la atención médica

☐ sí, pero tengo algunos problemas para acudir a servicios médicos

☐ no, porque tengo grandes dificultades para obtener servicios médicos

52. ¿Qué seguro médico tiene usted en Alemania?

☐ seguro alemán obligatorio o privado

☐ seguro para estudiantes

☐ seguro extranjero

☐ no tengo seguro médico
