# Supplementary material for: Knowledge, attitudes, behaviors, and serological status related to Chagas disease among Latin American migrants in Germany: A cross-sectional study in six German cities
Source: Front Cell Infect Microbiol. 2023 Jan 25;12:1047281. doi: 10.3389/fcimb.2022.1047281 (PMC9905718; doi:10.3389/fcimb.2022.1047281)
Supplement: Supplementary file 2 [file DataSheet_2.pdf]

Número de participante (llenado por el responsable del estudio)

# ElCiD – Proyecto alemán de Chagas
# Cuestionario sobre la enfermedad de Chagas en Alemania

**Prezado(a) Participante,**
**Um dos objetivos do estudo do Mal de Chagas é melhorar a atencao médica na Alemanha para imigrantes ou pessoas com risco de adquirir essa enfermidade. Para isso gostaríamos de obter algumas informacoes pessoais bem como sobre o conhecimento que tem sobre o Mal de Chagas. Nesse contexto da nossa proposta de assessoramento e e de testes do Mal de Chagas, lhe pedimos para responder algumas perguntas.**
**Essas informacoes serao confidenciais e analisadas separadamente dos dados pessoais (por exemplo nome, data de nascimento). A avaliacao será realizada exclusivamente para efeito de estatística. Dessa maneira nao haverá possibilidade de se ligar os dados às pessoas.**
**Desde já agradecemos a sua ajuda!**

**1. Sexo** ☐ **masculino** ☐ **feminino**

**2. Ano de nascimento** _______________

**3. País em que nasceu:** ________________ **4. Cidade em que nasceu:** ____________________

**5. Nacionalidade:** ______________________ **6. Idioma materno:**___________________

**7. Em que ano chegou na Europa?** ___________________________

**8. Qual a razao de sua vinda/mudanca para a Europa?**

☐ trabalho ☐ estudos ☐ matrimônio/ligacao afetiva ☐ familia vive na Europa
☐ outras razoes, que seriam (9) ____________________________________

**10. Em que país passou a maior parte de sua infância?** ________________________

**11. Em que cidade passou a maior parte de sua infância?** ___________________________

**12. Qual foi seu último domicilio antes de mudar para a Europa?**

país: ______________________________ cidade/regiao (13) :________________________

**14. Em que lugar da América Latina passou a maior parte da sua infância?**

☐ Em uma cidade (mais de 10.000 habitantes)
☐ No campo (menos de 10.000 habitantes)

**15. Seu último domicilio na América Latina foi....**

☐ Na cidade (más de 10.000 habitantes)
☐ No campo (menos de 10.000 habitantes)

**16. De que material era construida a casa onde passou a maior parte de sua infância?**

☐ pedra/concreto/cimento ☐ adobe/ barro ☐ madeira
☐ outros materiais

**17. De que material foi construida a casa onde morou por último antes de sua mudanca para Europa?**

☐ pedra/concreto/cimento ☐ adobe/ barro ☐ madeira
☐ outros materiais

**18. Alguma vez viu em sua casa um inseto chamado barbeiro (ou chinche, vinchuca, , chirimacha, pito, chichâ...)?**

☐ sim ☐ nao

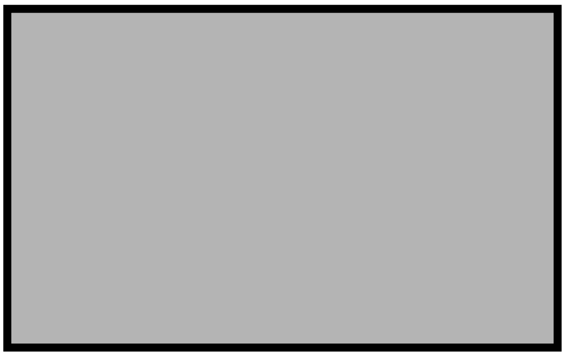

**19. Quantos filhos tem?** __________________________________

**20. Escolaridade?**

☐ nenhuma ☐ primário ☐ segundo-grau ☐ formacao técnica

☐ formacao universitária

**21. Qual o nível de escolaridade dos seus pais?**

☐ nenhum ☐ elementar ☐ segundo grau ☐ formacao técnica

☐ formacao universitária

**22. Tem conhecimento do Mal de Chagas em seu país?**

☐ sim ☐ nao

**23. Quais sao as moléstias que o Mal de Chagas pode causar?**
**Por favor mencione as três mais frequentes:**

1.________________________________________

2.________________________________________

3.________________________________________

**24. Como se transmite o Mal de Chagas?**
**(por favor marque todas as maneiras de transmissao que conhece)**

☐ **Por um barbeiro (vinchuca)**
☐ **Por relacoes sexuais**
☐ Por uma transfusao de sangue
☐ Por picada de mosquitos
☐ **Por um transplante de órgaos**
☐ **Por beber suco de cana**
☐ Ao bebê através da mae durante o parto
☐ **Por contato físico com pessoas infectadas**
☐ **Nao conhececo as formas de transmissao**

**25. Existem pessoas que se sentem bem apesar de estarem infectadas com o Mal de Chagas?**

☐ sim ☐ nao ☐ nao sei

**26. Alguém da sua familia direta tem ou teve o Mal de Chagas?**

☐ Sim, é meu(minha) (27) _________________________ ☐ nao ☐ nao sei

**28. Conhece alguém no lugar onde morou na América Latina que tem ou teve o Mal de Chagas?**

☐ sim ☐ nao

**29. Poderia se imaginar a doar sangue no futuro?**

☐ sim ☐ nao ☐ nao sei

**30. Já doou sangue?**

☐ sim, no seguinte país (31) ______________________ no ano de (32) __________

☐ nao

**33. Já recebeu uma transfusao de sangue?**

☐ sim, no seguinte país (34) ______________________ no ano de (35) __________

☐ nao

**36. Pode se imaginar sendo doador de órgaos após sua morte?**

☐ sim ☐ nao ☐ nao sei

**37. Já fez um teste para determinar o Mal de Chagas?**

☐ Sim, no ano (38) ____________________

(39) o resultado foi ☐ positivo ☐ negativo

☐ nao

**40. Caso o resultado tenha sido positivo, você recebeu algum tratamento para a doenca?**

☐ sim, no seguinte país (41): ________________no ano de (42) ____________________

com o seguinte medicamento (43) ________________________

☐nao

**44. Se você contraiu o Mal de Chagas, quais os sintomas você teve?**

1. ________________________________________________

2.________________________________________________

3.________________________________________________

**45. Tem alguma doenca de coracao?**

☐ sim ☐ **nao**

**46. Se tem alguma doenca de coracao, quais seriam os sintomas?**

1.____________________________________________________________

2.____________________________________________________________

3.____________________________________________________________

**47. Se tem uma doenca do coracao, qual seria o nome dessa enfermidade?**

1.________________________________________________________

2.________________________________________________________

3.________________________________________________________

**48. Sofre de alguma doenca do sistema digestivo** (estômago, esôfago, intestino)**?**

☐ sim ☐ nao

**49. Se sofre de uma doenca do sistema digestivo, quais seriam os sintomas?**

1.____________________________________________________________

2.____________________________________________________________

3.____________________________________________________________

**50. Se sofre de uma doenca do sistema digestivo, qual seria o nome dessa doenca?**

1.____________________________________________________________

2.____________________________________________________________

3.____________________________________________________________

**51. Você acha que o atendimento médico na Alemanha é suficiente?**

☐ sim, já que nao tenho problemas no acesso ao atendimento médico

☐ sim, mas tenho alguns problemas para acessar à alguns servicos médicos

☐ nao, porque tenho muita dificuldade em receber ao atendimento médico

**52. Qual o seguro saúde tem na Alemanha?**

☐ seguro alemao obrigatório ou privado

☐ seguro médico para estudantes

☐ seguro médico para estrangeiros

☐ nao tenho seguro médico
